# Funding Decisions for Newborn Screening: A Comparative Review of 22 Decision Processes in Europe

**DOI:** 10.3390/ijerph110505403

**Published:** 2014-05-19

**Authors:** Katharina Elisabeth Fischer, Wolf Henning Rogowski

**Affiliations:** 1 Hamburg Center for Health Economics, Universität Hamburg, Esplanade 36, 20354 Hamburg, Germany; 2 Helmholtz Zentrum München, German Research Center for Environmental Health, Institute of Health Economics and Health Care Management, Ingolstädter Landstr. 1, 85764 Neuherberg, Germany; E-Mail: rogowksi@helmholtz-muenchen.de; 3 Institute and Outpatient Clinic for Occupational, Social and Environmental Medicine, Clinical Center, Ludwig Maximilians University, Ziemssenstr. 1, 80336 Munich, Germany

**Keywords:** coverage, reimbursement, decision-making, internet survey, European Union, tandem mass spectronomy

## Abstract

Decision-makers need to make choices to improve public health. Population-based newborn screening (NBS) is considered as one strategy to prevent adverse health outcomes and address rare disease patients’ needs. The aim of this study was to describe key characteristics of decisions for funding new NBS programmes in Europe. We analysed past decisions using a conceptual framework. It incorporates indicators that capture the steps of decision processes by health care payers. Based on an internet survey, we compared 22 decisions for which answers among two respondents were validated for each observation. The frequencies of indicators were calculated to elicit key characteristics. All decisions resulted in positive, mostly unrestricted funding. Stakeholder participation was diverse focusing on information provision or voting. Often, decisions were not fully transparent. Assessment of NBS technologies concentrated on expert opinion, literature review and rough cost estimates. Most important appraisal criteria were effectiveness (*i.e.*, health gain from testing for the children being screened), disease severity and availability of treatments. Some common and diverging key characteristics were identified. Although no evidence of explicit healthcare rationing was found, processes may be improved in respect of transparency and scientific rigour of assessment.

## 1. Introduction

Population-based newborn screening (NBS) is considered as one public health strategy to prevent adverse health outcomes at infant age and improve the healthcare of rare disease patients [1,2]. Owing to the establishment of new technologies such as tandem mass spectrometry and identification of related gene mutations since the late 1990s and early 2000s, a variety of options have emerged to identify metabolic and genetic disorders at birth [3]. Given the public resources that can be spent for new screening programs are limited, choices need to be made about which of them should be conducted at the expense of public budgets.

Currently, the outcomes of these choices differ across Europe because the screening practice is heterogeneous [4]. To better understand the reasons for this heterogeneity and to provide advice about how decisions can be improved, descriptive information is needed about how the decisions were made. A further investigation of this question reveals a number of challenges. For example, there are different criteria which could play a role in decisions about screening programs [5]. Methods of health technology assessment (HTA) have been developed for developing scientific evidence about whether or not a decision criterion is met. To date, the decision outcomes can hardly be explained by this scientific evidence because HTA still has a limited impact on decision practice [6]. Furthermore, health care funding in Europe is typically organized within a national health service or a statutory health insurance. Decisions to include new interventions into the scope of publicly funded services are therefore made in the context of complex institutional procedures which may also have an impact on the decisions but which are not easy to describe [7,8,9]. For example, it has been put forward that besides the reasonableness of the decision criteria, also characteristics of the decision processes such as participation and transparency are relevant for fair decisions about health care resource allocation [10]. However, it is not straightforward to grade in a comparative manner to what extent different decision processes correspond with the fuzzy concept of “transparency”. The complexity of decision criteria and processes may additionally involve that perceptions about the characteristic values may differ so that it is difficult to obtain reliable information [11].

To obtain a comprehensive picture of decisions to fund new NBS programs, the objective of this study was to describe decision processes about new NBS programmes in Europe in a methodologically controlled manner. This involved a structured survey and an approach to validate the respondent’s answers.

## 2. Methods

Decisions that determine if and how a technology is included into the benefit basked of a health care payer can be analysed regarding several aspects, e.g., appraisal criteria, decision outcome, assessment methods or attributes of the decision process [12]. This study analysed these decisions from a process-oriented perspective. For this purpose, approaches have been developed to structure the characteristics of such decision processes at a national or regional level [7,8]. The conceptual framework by Rogowski elaborates the stylized steps that a decision passes through from emergence into markets to technology diffusion in clinical use [8]. It has been derived from expert opinions and literature search. Whilst covering a manageable number of elements, it inherits three properties that are suitable for analysing NBS decisions at European level: (1) It takes a broad view, describing all items relevant in the decision process. (2) It is generic to enhance an international comparison of processes. (3) It is based on information which is comparatively easy to observe such that its elements can be analysed for the different countries. It was therefore used in our analysis and structures the steps as follows:

− *Scope of decision making:* The process starts after the product has received marketing authorization or is known to have entered the scope of a payer.
− *Trigger:* As different decision bodies may coexist at national and regional level, the process of the responsible body needs to be triggered.
− *Participation:* There are different stakeholders who may be involved in the decision, e.g., manufacturers, service providers or patients.
− *Publication:* During the different steps, the decision-maker can disclose information about the process or its outcome.
− *Assessment:* The decision-maker applies some method of assessing the technology, e.g., by systematic literature review or expert opinion.
− *Appraisal:* A decision about whether to cover a technology is made based on an appraisal of certain criteria, e.g., a product’s effectiveness or cost-effectiveness.
− *Reimbursement:* In the case of a positive decision, a mechanism for reimbursement (*i.e.*, the level and mechanism of funding) has to be determined.
− *Management:* Besides funding the use of a technology, the payer may exert influence on service provision.

For this conceptual framework, a corresponding set of indicators for each step (see Table A1.for a description of the operationalization) has been developed [9]. The indicators were obtained from six case studies from the domain of cancer prevention, complemented by expert discussion. This allows structured comparison between decisions using pre-defined items in each step.

The conceptual framework and the set of indicators have been used for a survey of coverage decisions on NBS made between 2005 and 2009 of the EU 7th framework project HIScreenDiag (Grant No. 223533) [13]. Experts affiliated either to NBS providers or third party payers were approached via a scientific society (International Society of Neonatal Screening). They completed an internet survey on past decisions of NBS in their country/region they were familiar with by direct involvement or as an outside observer. The survey was conducted between August and December 2009. Respondents were asked for decisions made in the last five years to reduce recall bias. Forty-three respondents from 21 countries completed the questionnaire (response rate 70%). Respondents were contacted via an internet survey which included questions that covered the indicators of the structured scheme.

This study followed the principles of Good Scientific Practice issued by the German Research Foundation [14]. This includes the application of methods following the state of the art which was oriented at a textbook by Schnell *et al.*; for example, all respondents were offered confidentiality [11]. No approval by the responsible medical ethics board in addition to these standards was obtained because the survey addressed technical experts and decision makers with regard to reimbursement decisions. The study was not medical but socio-economic health services research. Neither patient nor any identifiable patient data was analysed. This kind of research was thus considered out of the scope of the World Medical Association (WMA) Declaration of Helsinki which addresses “medical research involving human subjects, including research on identifiable human material and data” [15]. 

We used the data set to elicit the key characteristics by the steps of decision-making. Plausibility and implications of the findings were discussed with the members of the HIScreenDiag project consortium to conclude in key characteristics of NBS decision-making for each step.

The survey included NBS conditions that have been considered for coverage for which a testing technology is available. We limited the scope of the study to blood spot screening. The decision could be made for an NBS programme that includes several conditions screened in one assay (e.g., introduction of MS/MS that covers several targets) or, expansion of an existing NBS programme by a condition, potentially involving the introduction of a new technology. Each target was considered as one decision for cost coverage. We disregarded all decisions that determine the exact technical implementation of an assay as these have been described previously [16].

A decision object was defined by screening method, tested condition, patient group and further specifics of the testing strategy. It could result in full, partial or no financial coverage. The study focused on screening for medium-chain acyl-CoA dehydrogenase deficiency (MCADD), cystic fibrosis (CF) and congenital adrenal hyperplasia (CAH). These conditions were identified in exploratory analyses as frequent decision objects [16]. If none of the conditions were identified, the research design was capable to include other case studies if respondents could not provide information about the above mentioned decisions.

The International Society for Neonatal Screening served as a platform to make contact with two experts per each EU member state and Switzerland involved in decision-making. From a total of 55 decisions, the 22 decisions from 11 countries that had been cross-validated by two experts were kept. Table 1 displays the included decisions by country which cover about 50% of the EU population. A first comparison of answers for same decisions showed that the inter-rater reliability was low. To ensure validity, data were compared for the decisions where two respondents provided answers. If deviations occurred, a Delphi procedure was applied. Here, we compared the answers of the two experts question by question. In case of deviations, we sent the diverging answers to the two experts again without disclosure of the other respondent’s name. The experts were asked for the reasons why their answers deviated and what they believed the correct answer was. The procedure was stopped when the percentage of conflicts remained in less than 5% of answers. Then, statements from the expert with the closest relation to the decision were considered, e.g. statements from payers were preferred to service providers.

For each indicator, we calculated the frequencies of categories. Data evaluation was performed with SAS Version 9.2 (SAS Institute, Cary, NC, USA). For the relevance of appraisal aspects, we considered the answers from both respondents.

**Table 1:** Coverage decisions on newborn screening (NBS) programmes in Europe considered.

Country (by population size)	Metabolic / genetic disorders considered in coverage decision
Germany	MCADD, CAH
France	CF
England	MCADD, Hb SS, CF
Romania	PKU, CH ^a^
Netherlands	MCADD, Hb SS
Czech Republic	CAH, CF
Hungary	MCADD, MSUD, GALT
Switzerland	MCADD
Denmark	MCADD, CAH
Slovenia ^b^	MCADD, CAH
Belgium: Region of Flanders	MCADD, BIO

^a^ Expansion of number of newborns screened; ^b^ Selective screening; Abbreviations: BIO, biotinidase deficiency; CAH, congenital adrenal hyperplasia; CF, cystic fibrosis; CH, congenital hypothyroidism; GALT, classical galactosaemia (galactose-1-phosphate uridyltransferase deficiency); Hb SS, sickle cell disorders; MCADD, medium-chain acyl-CoA dehydrogenase deficiency; MSUD, maple syrup urine disease; PKU, phenylketonuria.

## 3. Results and Discussion

For each step of the framework, we identified several characteristics which we summarized in Table 2. Frequencies of indicators for the steps scope of decision-making, trigger, publication, assessment, appraisal, reimbursement and management are displayed in Table 3. Results of the step participation are provided in Table 4. An overview by country is provided in Table A2.

### 3.1. Scope of Decision-Making

Decisions may depend on their scope, *i.e.*, whether they are made at individual, service provider, regional or national level. All decisions about NBS technologies were made by tax funded sources or the respective statutory health insurance. Except for the Flanders region in Belgium, decisions were made at national level (the UK National Screening Committee decision for England was considered as a national decision).

### 3.2. Trigger

Not all new technologies are subject to explicit coverage decisions. It is therefore important to consider how a decision process was triggered. Almost three fourth of decision processes were reported to be started after application of explicit criteria. 

**Table 2:** Characteristics of coverage decision processes of NBS programmes in Europe, according to 22 decisions obtained from expert survey**.**

Step of decision process	Characteristics
Scope of decision-making	Often not explicitly addressed by “typical” decision-making committees for financial coverage; In many countries, decisions were made at macro (*i.e.*, national) level.
Trigger	Start of decision processes predominantly after explicit specification of criteria.
Participation	Diverse participation of stakeholders; Service providers (laboratories) took high influence; Compared to pharmaceuticals, industry less involved; Stakeholders often involved in voting.
Publication	Information on decisions can hardly be validated via web- or document-search; Decision outcome not reported in about 10% of decisions; Stakeholder comments, HTA report, rationale for assessment question reported in less than 25% of decisions; Additionally some related information, but never full documentation of the process.
Assessment	Use of scientific evidence on effectiveness in 86% of decisions, but in 9% based on expert opinion only; Systematic review frequently indicated but often no possibility for validation; seven decisions with HTA; Assessment of cost predominantly based on cost-estimate.
Appraisal	High diversity of appraisal aspects; Health gain from testing for the children being screened, severity and availability of treatment for disease most relevant; Economic aspects less relevant; Lobbying activities had minor to no relevance.
Reimbursement	Predominantly positive, unrestricted funding; Different types of reimbursement: predominantly per test, insured, year.
Management	In at least one fifth of decisions no further reporting required to payer; Predominantly specific information about screening required.

**Table 3:** Overview of indicators from internet survey of decisions on NBS in European countries (*n* = 22).

Decision step	Indicator	Category	*n*	% (rounded)
Scope of decision-making	Type of third party payment	Tax funded	11	50%
		Statutory health insurance	11	50%
Trigger	Start of decision process	Explicit specification of criteria for trigger	16	73%
		Ad-hoc selection	6	27%
Publication	Reporting ^a^	Decision outcome	20	91%
		Decision rationale	7	32%
		Health technology assessment report	7	32%
		Attendance at or minutes of appraisal meeting	6	27%
		Stakeholder comments	3	14%
		Rationale for assessment question from scoping	2	9%
		No information available	2	9%
		Other	0	0%
	Transparency	Missing	11	50%
		Publication of decision and some supporting documents only	2	9%
		Decision and some supporting information	8	36%
		Selected relevant documentation	1	5%
		All process relevant documents	0	0%
		Full documentation	0	0%
Assessment	Assessment of effectiveness ^a^	Expert opinion	20	91%
		Systematic literature review	19	86%
		Other type of assessment	10	45%
		Quantitative meta-analysis of studies	2	9%
		No assessment of effectiveness	1	5%
	Assessment of costs/cost-effectiveness	No assessment of costs	2	9%
		Cost estimate	16	72%
		Cost-effectiveness analyses	4	18%
Appraisal	Effectiveness (health gain from testing)	Not relevant	2	9%
		Relevant	4	18%
		Strongly relevant	16	73%
	Severity of the disease	Not relevant	4	16%
		Relevant	7	32%
		Strongly relevant	11	52%
	Availability of treatment for disease	Not relevant	4	16%
		Relevant	9	41%
		Strongly relevant	9	43%
	Quality of evidence	Not relevant	6	25%
		Relevant	11	52%
		Strongly relevant	5	23%
	Expected costs	Not relevant	10	45%
		Relevant	7	34%
		Strongly relevant	4	20%
Appraisal	Cost-effectiveness	Not relevant	11	50%
		Relevant	7	30%
		Strongly relevant	4	20%
	Budget impact	Not relevant	9	39%
		Relevant	11	52%
		Strongly relevant	2	9%
	Effect on equitable access to health care	Not relevant	13	59%
		Relevant	8	36%
		Strongly relevant	1	5%
	Effectiveness (other benefit of know­ledge from testing)	Not relevant	15	70%
		Relevant	6	25%
		Strongly relevant	1	5%
	Lobbying by service provider(s)	Not relevant	13	57%
		Relevant	9	41%
		Strongly relevant	0	2%
	Lobbying activities by patients/patient representatives	Not relevant	11	50%
		Relevant	11	48%
		Strongly relevant	0	2%
	Scientific interest in gathering further evidence	Not relevant	18	80%
		Relevant	4	20%
		Strongly relevant	0	0%
	Lobbying activities by industry	Not relevant	22	98%
		Relevant	0	2%
		Strongly relevant	0	0%
	Lobbying by government	Not relevant	22	100%
		Relevant	0	0%
		Strongly relevant	0	0%
	Third payer’s concern for cost containment	Not relevant	22	100%
		Relevant	0	0%
		Strongly relevant	0	0%
Reimbursement	Decision outcome	Full cost coverage	18	82%
		Partial cost coverage	4	18%
		No coverage	0	0%
	Type of reimbursement (after decision) ^a^	Per test	15	68%
		Per year	6	27%
		Per covered individual	3	14%
		Per diagnosis related group based case	2	9%
		Other	0	0%
		Co-payment from insured	0	0%
	Mix of types of reimbursement	After decision	6	27%
Reimbursement	Type of reimbursement (before decision) ^a^	Other (including no reimbursement)	7	32%
		Per test	6	27%
		Co-payment from insured	4	18%
		Per year	3	14%
		Per diagnosis related group based case	2	9%
		Per covered individual	1	5%
Management	Information provision of service provider	At least number of reimbursed services	6	27%
		At least specific information about services	13	59%
		At least pre-authorization of services	3	14%

Note:^ a^ More than one was answer possible.

**Table 4:** Stakeholder participation (*n* = 22 coverage decisions).

Stakeholder Type of involvement *n* (%) ^a^	Service provider(s)	Payer	Patients	Government	Industry	Other
No involvement	1 (5%)	4 (18%)	7 (32%)	3 (14%)	20 (91%)	17 (77%)
Information provision ^b^	17 (77%)	6 (27%)	14 (64%)	5 (23%)	0 (0%)	8 (36%)
Appeal ^b^	1 (5%)	0 (0%)	1 (5%)	0 (0%)	0 (0%)	3 (14%)
Voting ^b^	2 (9%)	12 (55%)	1 (5%)	9 (41%)	1 (5%)	0 (0%)

Notes:^ a^ rounded; ^b^ More than one answer was possible.

Examples were availability of new research evidence (England), revision of screening criteria (The Netherlands) or, the right of proposal by expert groups (France). However, criteria did not become evident for most decisions although respondents were able to make specifications in the questionnaire.

### 3.3. Participation

Various types of stakeholders participated in decision-making. Table 4 provides an overview of participation by stakeholders and their type of involvement. Involvement by service providers, the payer and government was most frequent. Neither government nor payers were involved in Romania. Besides public authorities, service providers were strongly involved. In a number of countries these are concentrated to few national centres, e.g., in Germany, Denmark or Hungary [4]. Compared to pharmaceuticals where manufacturers often need to submit applications, respondents indicated that industry was explicitly involved only in Switzerland and France [17]. In the Danish decisions and in Romania for expansion of screening coverage for phenylketonuria (PKU), neither involvement by industry nor service providers was reported. Involvement by patients cannot be disregarded as participation was reported in 68% of decisions. 

Stakeholder involvement concentrated on providing information relevant for the decision and voting on the final outcome. Information was mainly prepared by service providers and patients. The payer and government were those who were involved in voting most frequently.

### 3.4. Publication

Transparency is frequently demanded to ensure fair decision-making. For the step “publication”, this was described by the types of documents provided and the degree of transparency which we derived from the type of information. Publication of the decision outcome, rationale, a related HTA report and minutes from the appraisal meeting were reported most frequently. In the decisions in the Czech Republic, information on the decision outcome was not provided publicly. Stakeholder comments and the rationale for the assessment question from scoping were reported in less than 20% of decisions. An HTA report was provided in Denmark, The Netherlands and England. There was no decision with complete documentation or at least including process relevant documents. Validation of reported documentation was difficult, because we frequently could not obtain related information on websites. No information was provided in the decisions made in Slovenia. In 50% of decisions only one type of documentation was published.

### 3.5. Assessment

The principles of evidence-based healthcare require that decisions are based on best available evidence. This was considered in the step of ‘assessment’. The survey investigated standard elements of technology assessment in terms of effectiveness and cost-effectiveness. According to the respondents, effectiveness was predominantly assessed by a systematic literature review. However, we could not validate this for every decision. From 19 decision processes reporting an own review, information on the search strategy was obtained for ten cases. In other six cases, we could not identify a systematic review. Despite of recent economic evaluations on NBS, we found that the assessment of costs/cost-effectiveness was based on a cost estimate rather than a full economic evaluation in 73% of decisions [18]. Cost-effectiveness analyses have been conducted in 18% of decisions. In Slovenia, costs were not assessed.

### 3.6. Appraisal

Ultimately, the decision maker’s choices in favor or agains funding a technology involve value judgments. According to Daniels and Sabin, the principles of fair allocation of scarce health care resources require that decision makers only apply those aspects in decision making for which all fair-minded parties agree that they are relevant for appropriate care under resource constraints [19]. Appraisal criteria which are frequently cited in the literature contain concerns about the intervention’s effectiveness and cost-effectiveness, severity of disease or equity considerations [20]. The appraisal criteria applied in this survey were based on a previous review and qualitative case study [9]. As an indicator or criteria which may meet these criteria to a lower extent, the survey additionally included particular interests/lobbying activities of stakeholders.

By far, effectiveness in terms of health gain from testing (73% of decisions), disease severity (52%), and the availability of a treatment (43%) were most frequently rated as strongly relevant. Aspects related to economic criteria were considered in fewer decisions (cost-effectiveness, expected costs (each 20%) and budget impact (9%)). Although stakeholders participated in all processes to some extent, stakeholder interests were reported to have minor influence during appraisal. Amongst these, patients’ interests appeared most relevant. They were strongly relevant in the decision on medium-chain acyl-CoA dehydrogenase deficiency (MCADD) screening in England. Service provider interests played a role in the decision on galactosaemia screening in Hungary.

### 3.7. Reimbursement

Positive decisions do not inevitably mean that a technology is fully funded. Instead, different reimbursement payment schemes may co-exist within one health care system for different types of technologies, e.g., capitation-based reimbursement, fee for service. These may deviate from the actual cost of a medical service. Four decisions resulted in partial coverage (*i.e.*, only a part of the costs of screening are funded by the payer), all other programmes were granted full coverage. None of the technologies were excluded from funding. At least 70% of technologies have received funding to some extent before the final decision was made. For example, pilot studies were supported in certain regions or, the screening was provided from special budgets. For 18% of technologies services could be obtained through co-payments before the decision. After the decisions, reimbursement was predominantly provided per test or as a fixed budget while capitation-based payment or reimbursement within a DRG-catalogue had minor relevance.

### 3.8. Management

To complement the reimbursement regime, payers may exert influence on service provision by other means, e.g. by implementing requirements to monitor service provision in more detail than just counting the number of services. For about 60% of decisions, the payer has introduced requirements to report programme information. For example, outcomes of the NBS programme in Germany are disseminated biannually [21]. Pre-authorization was only required from the payer before the test may be conducted in the Flanders region (Belgium) and for expansion of PKU screening in Romania.

## 4. Discussion

### 4.1. Interpretation of Results

For the 22 decision processes on NBS programmes in Europe, we identify a number of common and diverging characteristics. Decisions were typically made at national healthcare system level. Processes were rather intransparent in terms of the documents provided. Effectiveness of the screening strategies was predominantly assessed by expert opinion and systematic reviews. Of note, the use and relevance of systematic reviews may be over-estimated from our results as we could not reassure the presence of a systematic review in six of 20 decisions. 

The lower evidence standards for effectiveness and cost-effectiveness compared with pharmaceuticals (e.g., randomized controlled trials) may be due to the fact that high levels of evidence are typically difficult to meet for NBS technologies. The main reasons are that many disorders account as orphan diseases, the natural history of the diseases is not well characterized, an effective treatment may not be available or, treatment effects are highly uncertain [22]. For example, for CF, the evidence on effectiveness is ambiguous [5].

Economic criteria that consider a technology’s cost, cost-effectiveness or budget impact seemed to have minor relevance. For many programmes, this may be due because of two reasons [23]: (1) The direct costs of additional screening tests are relatively low. (2) Considerable uncertainty is often involved in the effects that are generated by the available treatment options after screening such that economic considerations are overruled. Thus, it seems that not only have economic criteria played little role but also the evidence of effectiveness has been interpreted more positively than a conservative application of the principles of evidence-based medicine would imply. Moreover, despite discussions about scarce healthcare resources, our data do not reveal examples of healthcare rationing: although our survey design allowed for negative decision outcomes, all funding decisions were positive and economic considerations were reported to be of minor relevance in appraisal.

The decision processes were less homogenous in the steps of participation, reimbursement and the relevance of appraisal criteria. These steps appear to especially reflect the diversity of healthcare systems in terms of the funding arrangements within which NBS is provided, the establishment of stakeholder roles and the diverging value judgements of policy-makers. 

The industry was hardly involved. One explanation for this could be that in the eight decisions made on MCADD, the treatment does not involve costly pharmaceuticals, but mainly consists of avoiding staying without food for a long time. On the contrary, patients seemed to have sufficient possibilities to at least provide information. 

### 4.2. Limitations

The study is faced with a number of limitations which also reveal difficulties with characterizing the apparently simple topic of funding decisions in a comparative manner. Naturally, one would assume that decisions are made when the question arises whether a service should be implemented. Given that all assessed decisions were positive, formal decisions may rather have been made for confirmatory purposes after a medical or political decision in favour of screening had already been made, e.g., by funding a pilot stage of the programme. The assessment of the process step “trigger” appears to capture this to a very limited extent. Future studies should explore the shape and role of the process of technology implementation before a formal coverage decision [21]. Negative “pre-decisions” in the face of a positive funding decision may be identified for example by comparing conditions included in pilot tests with those in the final screening panel. For example, after a pilot phase in the German state of Bavaria between 1999 and 2004, not all conditions were recommended for universal screening under statutory health insurance [24].

Our results are limited by the small number of decisions that were included. This restricted statistical analysis to counting frequencies instead of multivariate analyses. A larger sample size and multivariate approaches would be needed to control for clustering effects by country or disease and to determine the impact of the steps of decision-making on the criteria used in appraisal or on decision outcomes. First evidence on the influences of the process steps has been provided for a sample of coverage decisions on health technologies not being restricted to a certain disease area. It indicated that rigorous assessment and stakeholder participation promoted reasonable decision-making whilst transparency did not have an influence [25]. Also, decision outcomes of coverage decisions appeared to be influenced mostly by the use of evidence rather than the degree of transparency or participation [26]. 

However, the small number of included was also because only information validated during a Delphi process was included which appeared to be an important methodological approach to obtain reliable results. Before the Delphi procedure, the inter-rater reliability for the rather simple, structured questions was very low. An explanation for this could be the poor documentation so that recalling the actual course of decision processes was difficult. We only included data which were validated by two respondents as the Delphi method allowed increasing the concordance between respondents by 50% to about 95%. However, this method was very lengthy. 

The results do not reflect decision-making of member states in the South of Europe (e.g., Spain, Italy) as no answers were obtained from these regions that have been evaluated by two respondents. Finally, we have to note that only decisions made between 2005 and 2009 were included. After this point in time, decisions on NBS were made in a number of countries, for example for MCADD in France [27].

### 4.3. Implications for Further Research

In relation to existing literature on NBS policies, this survey is among the first that emphasizes the process of funding decisions. Previous studies have dealt with screening algorithms and technical description of the programmes [4,16,28,29]. Further research presents case studies of selected countries or disorders [30,31,32,33]. Other studies focus on the definition of appraisal criteria from which our approach departed [5,34,35]. While most studies refer to the Wilson and Jungner criteria which are dedicated to appraisal of screening programmes [3,5], we use a set of general clinical, economic and other ethical criteria that are comparable across different types of intervention or healthcare systems. Even if this study used a validated framework for describing the decision processes and the criteria used [9], the description and analysis of health care funding decisions remains a young area of health services research. Further work is necessary to better describe and understand these choices which determine the health services available to patients.

Particular evaluation frameworks for genetic tests have been developed such as the ACCE framework that provides a tool to evaluate analytic validity, clinical validity, clinical utility and ethical, legal and social issues for genetic testing have been developed [23,36,37]. However, as the technological options available to payers rise, there is a tendency that coverage is evaluated and determined by a single body that deals with the majority of health services, e.g., in France [38]. In parallel, economic considerations appear to gain in importance such that NBS will likely need to show cost-effectiveness which goes beyond the scope of the established criteria for screening technologies [39]. Besides further work regarding the positive description and analysis of decisions about health care funding, also further work about relevant evaluation frameworks is needed to ensure that the evaluation frameworks appropriately account for fairness concerns. Also, the appropriate balance between generic frameworks to facilitate consistent decisions across all areas of health care and condition-specific frameworks to facilitate sensitivity for aspects particularly relevant for clinical areas such as NBS are needed.

There are attempts to harmonize decision processes for new NBS programs across Europe [40,41]. Also in the United States, national evidence based guidance provided in the core panel by the American College of Medical Genetics (ACMG) led to harmonization of screening panels at state level and reducing inequity [42]. This study illustrates potential areas of improvement which might benefit from standardization on an EU level, for example regarding the limited transparency or the fact that decisions were not always based on evidence a proponent of evidence-based medicine might expect. However, it also illustrates that currently, much remains to be learnt about how decisions to fund new NBS programs are made today. Further research about the status quo thus be an important starting point to ensure that new decision frameworks do indeed build upon the best practices rather than repeating regional or national mistakes on the level of the EU or the US.

## 5. Conclusions

This study elicits key characteristics of cost coverage decision-making on expanded population-based NBS programmes across Europe by the steps of decision processes. Despite variations, most payers of public healthcare systems have defined mechanisms to assess and appraise NBS. Characteristics show that most processes allow diverse stakeholder participation and appraise technologies most frequently regarding their effectiveness, the disease severity and treatment availability. We identify potential for improvement in transparency of decision-making and the use of evidence regarding cost-effectiveness. Despite discussions about scarce healthcare resources, explicit healthcare rationing was not identified. 

## Appendix

**Table A1:** Operationalization of steps of conceptual framework.

Decision Step	Operationalisation	Indicator(s)
Scope of decision-making	Classification of type of health care system funding.	*Type of third party payment* - Tax funded - Statutory health insurance
Trigger	Information whether decision process was started by definition of explicit criteria for selection of technology or, technology was selected ad-hoc.	*Start of decision process* - Ad-hoc selection - Explicit specification of criteria for trigger
Participation	Number and types of different stakeholders being formally involved and their involvement.	*Types of stakeholders involved:* - Service provider(s) - Payer - Government - HTA group or agency - Patients/patient representatives - Industry - Academia - Other stakeholder(s) *Level of involvement of formally participating stakeholders:* - Information provision - Voting - Appeal
Publication	Number and types of different documents that have been published during or after the decision process.	*Types of documentation accessible to public during/after decision process* - Attendance at or minutes of appraisal meeting - Decision rationale - Decision outcome - Stakeholder comments - Rationale for assessment question from scoping - No information available - Other
Assessment	Methods that were used for assessment of effectiveness and costs/cost-effectiveness.	*Assessment of effectiveness:* - No assessment of effectiveness - Expert opinion - (Systematic) literature review - Quantitative meta-analysis of studies *Assessment of cost-effectiveness:* - No assessment of cost-effectiveness - Cost estimate - Cost-effectiveness analyses
Appraisal	Aspects that were considered relevant or strongly relevant for the decision outcome	*Aspects relevant for outcome of decision* Effectiveness (other benefit of knowledge from testing) Severity of the disease Availability of treatment for disease Quality of evidence Expected costs Cost-effectiveness Budget impact Effect on equitable access to health care Effectiveness (other benefit, e.g., knowledge of test result) Lobbying by service provider(s) Lobbying by patients/patient representatives Scientific interest to gather further evidence Lobbying by industry Lobbying by government Third payer’s concern for cost containment
Reimbursement	Different mechanisms of reimbursement may have been before and after the decision.	*Decision outcome* - Full cost coverage - Partial cost coverage - No cost coverage *Type of reimbursement (before decision)* - Per test - Per year - Per covered individual - Per diagnosis related group based case - Other - Co-payment from insured *Type of reimbursement (after decision)* - Per test - Per year - Per covered individual - Per diagnosis related group based case - Other - Co-payment from insured
Management	After the decision, the payer may have requested regulations for implementation of the technology	*Information provision of service provider* - Number of reimbursed services - Specific information about services - Pre-authorization of services

**Table A2:** Results by country.

Country	Decision	Payer	Deciding committee	Trigger	Assessment (Effectiveness/Cost/CE)	Participation	Publication	Appraisal	Decision outcome	Reimbursement (after decision)	Reimbursement before decision)	Management
Germany	MCADD, tandem mass spectrometry as first tier	SHI	Federal Joint Committee	Explicit specification of criteria	Effectiveness: Expert opinion Cost/CE: Rough cost estimate	Information provision: patients, academia; voting: provider(s), payer; other type of participation: government	Outcome	Effectiveness: health gain, evidence	Partial cost coverage	Per insured	Per test	Number of services, specific information
Germany	CAH, immunoassays	SHI	Federal Joint Committee	Explicit specification of criteria	Effectiveness: Expert opinion Cost/CE: Rough cost estimate	Information provision: patients, academia; voting: provider(s), payer; other type of participation: government	Outcome	Effectiveness: health gain, treatment	Partial cost coverage	Per insured	Per test	Number of services, specific information
France	CF, immunoreactive trypsin test as first tier	SHI	No separate institution	Explicit specification of criteria	Effectiveness: Expert opinion, Systematic literature review Cost/CE: CE without guidelines	Information provision: provider(s), payer, patients;	Appraisal meeting, outcome, rationale	lobbying by: service providers, patient(s)	Full cost coverage	Per insured, per year	.	Number of services, specific information
England	CF, immunoreactive trypsin test as first tier	NHS	National Screening Committee	Explicit specification of criteria	Effectiveness: Expert opinion, Systematic literature review Cost/CE: Formalized cost estimate	Information provision: provider(s), government, patients, academia, other type of stakeholder; other type of participation: provider(s), government, patients, academia, other type of stakeholder	Appraisal meeting, outcome, HTA report, stakeholders	Effectiveness: health gain, cost,CE, lobbying by: patient(s)	Full cost coverage	Per test, per year	Other type of reimbursement	Number of services, Specific information,
England	Sickle cell disorders (tandem mass spectrometry)	NHS	National Screening Committee	Explicit specification of criteria	Effectiveness: Expert opinion, Systematic literature review Cost/CE: Formalized cost estimate	Information provision: provider(s), government, patients, academia, other type of stakeholder; other type of participation:	Appraisal meeting, outcome, HTA report, stakeholders	Effectiveness: health gain, cost,CE, treatment, lobbying by: patient(s)	Full cost coverage	Per test, per year	Other type of reimbursement	Number of services, specific information
England	Sickle cell disorders (tandem mass spectrometry)	NHS	National Screening Committee	Explicit specification of criteria	Effectiveness: Expert opinion, Systematic literature review Cost/CE: Formalized cost estimate	provider(s), government, patients, academia, other type of stakeholder	Appraisal meeting, outcome, HTA report, stakeholders	Effectiveness: health gain, cost,CE, treatment, lobbying by: patient(s)	Full cost coverage	Per test, per year	Other type of reimbursement	Number of services, specific information
England	MCADD, tandem mass spectrometry as first tier	NHS	National Screening Committee	Explicit specification of criteria	Effectiveness: Expert opinion, Systematic literature review Cost/CE: Formalized cost estimate	Information provision: provider(s), government, patients, academia, other type of stakeholder; other type of participation: provider(s), government, patients, academia, other type of stakeholder	Appraisal meeting, outcome, HTA report, stakeholders	Effectiveness: health gain, cost,CE, treatment, lobbying by: patient(s)	Full cost coverage	Per test, per year	Other type of reimbursement	Number of services, specific information
Romania	CH, expansion of number of infants screened	NHS	No separate institution	Ad-hoc selection	Effectiveness: Expert opinion, Systematic literature review Cost/CE: Rough cost estimate	Information provision: provider(s); other type of participation: government	No info during decision Outcome, Stakeholders,	Budget impact, treatment, lobbying by: patient(s)	Partial cost coverage	Per year	Per year	Number of services, specific information
Romania	Phenylketonuria, guthrie test, expansion of number of infants screened	NHS	No separate institution	Ad-hoc selection	Cost/CE: Formalized cost estimate	Information provision: patients; appeal: patients; voting: government;	Outcome	Budget impact, treatment, lobbying by: patient(s)	Partial cost coverage	Per year	Per insured	Pre-authorization
Netherlands	MCADD, tandem mass spectrometry as first tier	SHI	National Health Council	Explicit specification of criteria	Effectiveness: Expert opinion, Systematic literature review Cost/CE: Rough cost estimate	Information provision: provider(s), government, patients, academia, other type of stakeholder; voting: government;	HTA report, scoping, stakeholders, outcome, rationale	Effectiveness: health gain	Full cost coverage	Per test	co-payment	Number of services, specific information
Nether-lands	Sickle cell disorders (high-performance liquid chromatography)	SHI	National Health Council	Explicit specification of criteria	Effectiveness: Expert opinion, Systematic literature review Cost/CE: Rough cost estimate	Information provision: provider(s), government, patients, academia, other type of stakeholder; voting: government;	HTA report, scoping, stakeholders, outcome, rationale	Effectiveness: health gain, lobbying by: patient(s)	Full cost coverage	Per test	co-payment	Number of services, specific information
Czech Republic	CAH	SHI	Ministry of Health	Explicit specification of criteria	Effectiveness: Expert opinion, Systematic literature review Cost/CE: Rough cost estimate	Information provision: provider(s), academia; appeal: provider(s), academia; voting: payer, government;	Appraisal meeting, outcome, rationale	Effectiveness: health gain, evidence, lobbying by: service providers	Full cost coverage	Per test	Per test	Number of services, specific information
Czech Republic	CF, immunoreactive trypsin test as first tier	SHI	Ministry of Health	Explicit specification of criteria	Effectiveness: Expert opinion, Systematic literature review Cost/CE: Rough cost estimate	Information provision: provider(s), academia,appeal: provider(s), academia; voting: payer, government;	Appraisal meeting, outcome, rationale	Effectiveness: health gain, lobbying by: service providers	Full cost coverage	Per test	Per test	Number of services, specific information
Hungary	MCADD, tandem mass spectrometry as first tier	SHI	No separate institution	Explicit specification of criteria	Effectiveness: Expert opinion, Systematic literature review Cost/CE: CE in line with guidelines	Information provision: provider(s), payer, patients, academia, other type of stakeholder; appeal: other type of stakeholder; voting: payer, government;	Outcome	CE, lobbying by: service providers, patient(s)	Full cost coverage	Per test	Other	Number of services
Hungary	Galactosaemia (Photometric or fluorimetric enzyme assays, biochemical testing)	SHI	No separate institution	Explicit specification of criteria	Effectiveness: Expert opinion, Systematic literature review Cost/CE: CE in line with guidelines	Information provision: provider(s), payer, patients, other type of stakeholder; appeal: other type of stakeholder; voting: payer, government;	Outcome	Effectiveness: health gain, lobbying by: service providers, patient(s)	Full cost coverage	Per test	Per test	Number of services
Hungary	Maple sirup urine disease, tandem mass spectrometry as first tier	SHI	No separate institution	Explicit specification of criteria	Effectiveness: Expert opinion, Systematic literature review Cost/CE: CE in line with guidelines	Information provision: provider(s), payer, patients, academia, other type of stakeholder; appeal: other type of stakeholder; voting: payer, government;	Outcome	CE	Full cost coverage	Per test	Other	Number of services
Switzer-land	MCADD, tandem mass spectrometry as first tier	SHI	Bundesamt für Gesundheit (Ministry of Health)	Explicit specification of criteria	Effectiveness: Expert opinion, Systematic literature review Cost/CE: Formalized cost estimate	Information provision: provider(s); voting: payer, government, patients, Industry, academia;	Outcome	Effectiveness: health gain, effectiveness: other benefit	Full cost coverage	Per test	Per test	Number of services
Denmark	MCADD, tandem mass spectrometry as first tier	NHS	National Board of Health	Explicit specification of criteria	Effectiveness: Expert opinion, Systematic literature review Cost/CE: Formalized cost estimate	Information provision: payer, patients, academia, HTA agency; other type of participation: provider(s), government, HTA agency	Outcome	Effectiveness: health gain, evidence, treatment	Full cost coverage	Per test	Other	Number of services, specific information
Denmark	CAH (measurement of 17alpha-hydroxyprogesterone)	NHS	National Board of Health	Explicit specification of criteria	Effectiveness: Expert opinion, Systematic literature review Cost/CE: Formalized cost estimate	Information provision: payer, patients, academia, HTA agency; other type of participation: provider(s), government, HTA agency	Outcome, rationale	Effectiveness: health gain, evidence, treatment	Full cost coverage	Per test	Other	Number of services, specific information
Slovenia	MCADD selective screening	NHS	No separate institution	Ad-hoc selection	Effectiveness: No assessment of effectiveness Cost/CE: No assessment of costs	Information provision: provider(s), academia; voting: payer;	No information available	Effectiveness: health gain, treatment	Full cost coverage	Per DRG	Per DRG	Number of services
Slovenia	CAH selective screening (measurement of 17alpha-hydroxyprogesterone)	NHS	No separate institution	Ad-hoc selection	Effectiveness: Expert opinion, Systematic literature review Cost/CE: No assessment of costs	Information provision: provider(s), academia; voting: payer;	No information available	Effectiveness: health gain, treatment	Full cost coverage	Per DRG	Per DRG	Number of services
Belgium-Flanders	MCADD, tandem mass spectrometry as first tier	NHS	No separate institution	Ad-hoc selection	Effectiveness: Expert opinion, Systematic literature review Cost/CE: Formalized cost estimate	Information provision: provider(s); voting: payer; other type of participation: government	Outcome	Effectiveness: health gain, treatment, lobbying by: service providers	Full cost coverage	Per test	Per year, co-payment	Number of services, specific information, pre-authorization
Belgium-Flanders	Biotinidase deficiency (colorimetric or fluorimetric detection methods)	NHS	No separate institution	Ad-hoc selection	Effectiveness: Expert opinion, Systematic literature review Cost/CE: Formalized cost estimate	Information provision: provider(s); voting: payer; other type of participation: government	Outcome	Effectiveness: health gain, treatment, lobbying by: service providers	Full cost coverage	Per test	Per year, co-payment	Number of services, specific information, pre-authorization

Abbreviations: CAH: Congenital Adrenal Hyperplasia; CE: cost-effectiveness; CF: cystic fibrosis; CH: Congenital hypothyroidism; DRG: Diagnosis-related group; HTA: Health Technology Assessment; MCADD: Medium-chain acyl-CoA dehydrogenase deficiency; NHS: national health service; SHI: statutory health insurance;
